# Specific domain V reduction of beta-2-glycoprotein I induces protein flexibility and alters pathogenic antibody binding

**DOI:** 10.1038/s41598-021-84021-2

**Published:** 2021-02-25

**Authors:** Ina Buchholz, Thomas McDonnell, Peter Nestler, Sudarat Tharad, Martin Kulke, Anna Radziszewska, Vera M. Ripoll, Frank Schmidt, Elke Hammer, Jose L. Toca-Herrera, Anisur Rahman, Mihaela Delcea

**Affiliations:** 1 Institute of Biochemistry, University of Greifswald, Greifswald, Germany; 2 ZIK HIKE, University of Greifswald, Greifswald, Germany; 3 Division of Biochemical Engineering, Bernard Katz Institute, University College London, London, UK; 4 Institute of Physics, University of Greifswald, Greifswald, Germany; 5 Institute for Biophysics, University of Natural Resources and Life Sciences Vienna, Vienna, Austria; 6 Centre for Adolescent Rheumatology Versus Arthritis at UCL, UCLH, GOSH, London, UK; 7 Division of Medicine, Centre for Rheumatology, University College London, London, UK; 8 Interfaculty Institute for Genetics and Functional Genomics, University of Greifswald, Greifswald, Germany; 9 Proteomics Core, Weill Cornell Medicine-Qatar, Doha, Qatar; 10 DZHK (German Centre for Cardiovascular Research), Partner Site Greifswald, Greifswald, Germany

**Keywords:** Biochemistry, Biophysics

## Abstract

Beta-2-glycoprotein I (β2GPI) is a blood protein and the major antigen in the autoimmune disorder antiphospholipid syndrome (APS). β2GPI exists mainly in closed or open conformations and comprises of 11 disulfides distributed across five domains. The terminal Cys288/Cys326 disulfide bond at domain V has been associated with different cysteine redox states. The role of this disulfide bond in conformational dynamics of this protein has not been investigated so far. Here, we report on the enzymatic driven reduction by thioredoxin-1 (recycled by Tris(2-carboxyethyl)phosphine; TCEP) of β2GPI. Specific reduction was demonstrated by Western blot and mass spectrometry analyses confirming majority targeting to the fifth domain of β2GPI. Atomic force microscopy images suggested that reduced β2GPI shows a slightly higher proportion of open conformation and is more flexible compared to the untreated protein as confirmed by modelling studies. We have determined a strong increase in the binding of pathogenic APS autoantibodies to reduced β2GPI as demonstrated by ELISA. Our study is relevant for understanding the effect of β2GPI reduction on the protein structure and its implications for antibody binding in APS patients.

## Introduction

Beta-2 Glycoprotein I (β2GPI) is a five-domain glycoprotein is comprised of 326 amino acids^1^. The C-terminal Domain V (DV) is responsible for the ability of β2GPI to bind to cell surfaces. DIII and DIV are highly glycosylated with 4 or 5 glycan sites^2–4^. The beta-sheet rich domains are separated by flexible random coil regions that allow the protein to exhibit two major conformational states; the closed (circular) conformation and open (linear) conformation. The circular form was visualized by Agar et al*.*^5^, while the linear conformation was shown by X-ray crystal structure analysis^1,6^. A potential S-shaped conformation, which may represent an intermediate form, has been hypothesized^7,8^.

β2GPI is the major antigen in the autoimmune disorder antiphospholipid syndrome (APS)^9–13^. APS has an incidence of 2 per 100,000 people per year^14^ and is a leading cause of recurrent miscarriage and of strokes in patients under 50 years old. The major epitopic region of β2GPI, R39-G43, resides in DI^15–19^. Antibodies to this region have been shown to be pathogenic in mouse models and correlate well with thrombosis and pregnancy morbidity in patients^13,17,19–21^. It is hypothesized that in the circular form, this region in DI is hidden, giving the effect of a cryptic epitope^5,22^.

β2GPI has a number of physiological functions in the healthy body, which have been recently reviewed^23^, including both up- and down-regulation of the complement and coagulation cascades, a role in LPS scavenging^24^ and in prevention of melanoma spread^25^. The ability to act within two cascades in opposing manners is relatively unique to β2GPI.

Although the role of post-translational modifications in altering the structural conformation of β2GPI has been investigated^26,27^, the potential relationship between alternative structures of β2GPI and the different functions of the molecule is not understood fully.

In this paper, we examine the effect of disulfide reduction on the structure of β2GPI. All five domains of β2GPI contain two structural disulfides with little surface exposure. DV alone has a third disulfide (Cys288/Cys326) which is allosteric, generally associated with a structural change of proteins^28^. As shown by Ioannou et al*.*^29^, β2GPI includes different cysteine redox states of the Cys288/Cys326 disulfide bond under pseudo-physiological conditions^27^. Protein redox states have also been shown to be of interest in the pathogenesis of APS^30,31^, and in the production of pathogenic anti-β2GPI antibodies^31^. Studies looking at the terminal disulfide in DV have been conducted showing an association with the production of anti-DI antibodies^32–34^. However, the effects of reduction on the structural conformation of β2GPI have not been fully elucidated. This study aims to fully characterize by spectroscopic and imaging techniques the effect of specific reduction of Cys288/Cys326 on the structure of β2GPI, and its implications for binding of antibodies from patients with APS.

## Results

### Enzymatic reduction of β2GPI mainly targets the Cys288/Cys326 disulfide bond

**Figure 1:**
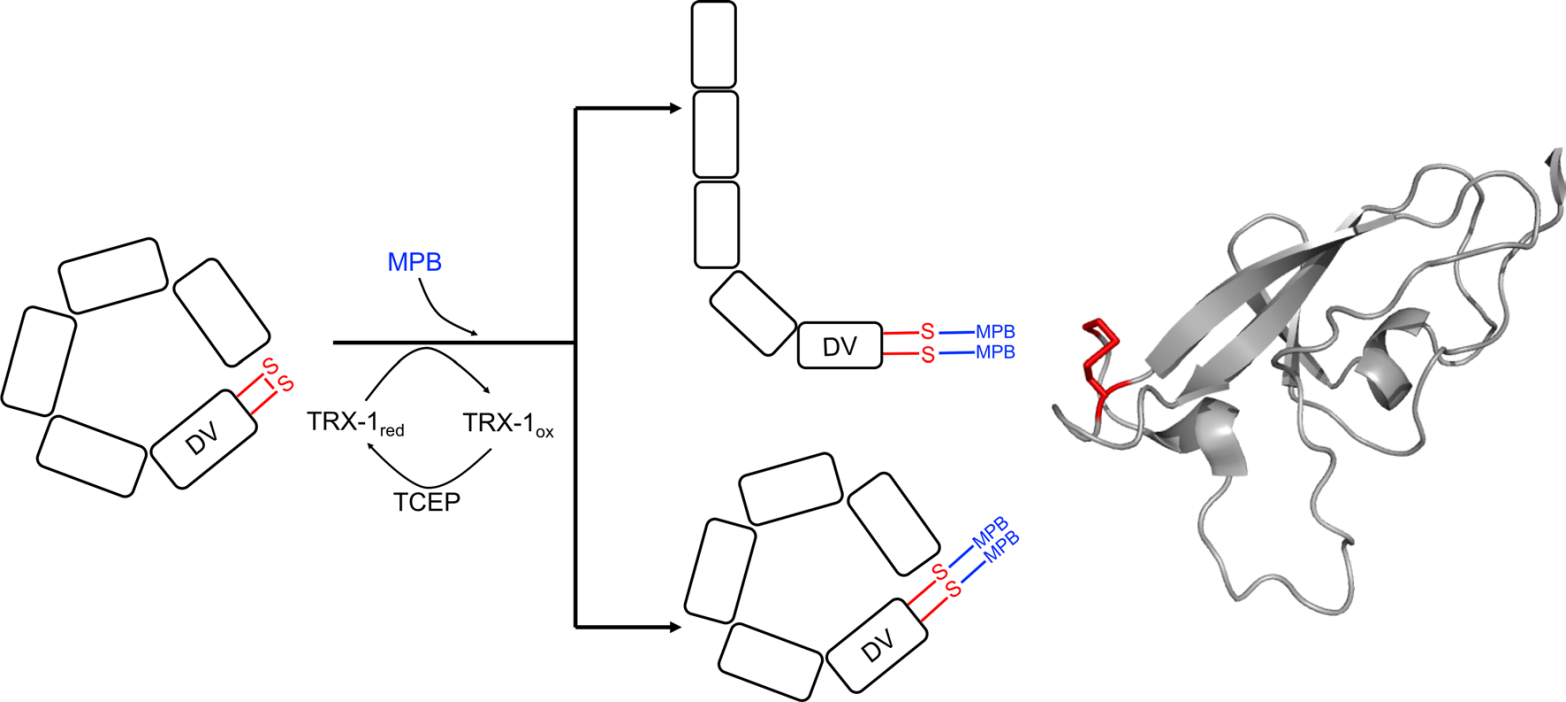
Left panel: schematic of the hypothesised reaction on circular β2GPI, highlighting the intact disulfide bond in the fifth domain (DV). The protein undergoes selective, enzymatic driven reduction by TRX-1 (recycled by TCEP) and liberates the two thiols, which are subsequently labelled by MBP to avoid re-oxidation. All reaction steps were performed under argon atmosphere at 25 °C in HBS buffer (pH 7.4) with mild shaking. 50 µM TRX-1 was reduced by 50 µM TCEP in a total volume of 100 µL HBS buffer for 1 h. 900 µL HBS buffer and 0.2 µM β2GPI were added and incubated for 1 h. 15 µM MPB were added for 15 min to label free thiols. Finally, 200 µM reduced GSH was used to quench unbound MPB (15 min). Right panel: C-terminal, fifth domain (DV) of β2GPI with the disulfide Cys288/Cys326 highlighted in red.

Specific reduction of β2GPI was carried out by TRX-1 as described in the “Methods” section (Fig. 1) and purification was achieved by SEC. To investigate the redox state of the protein, an Ellman’s reagent assay was used to quantify free thiols within untreated β2GPI (Supplementary Fig. S1). Although β2GPI has 22 cysteine residues, untreated protein gave a ratio of 0.19 ± 0.02:1 thiols per protein, indicating that the majority of protein’s thiols are in oxidized state.

**Figure 2:**
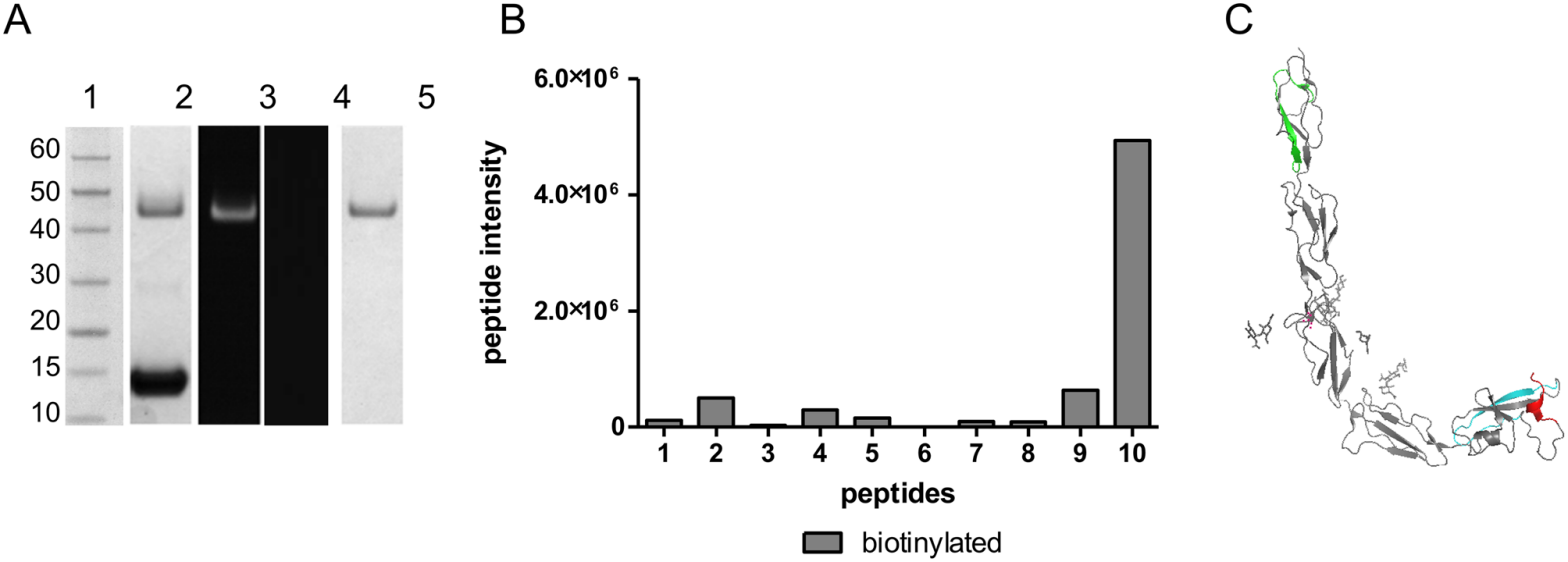
(**A**) SDS-PAGE and Western blot analyses (full-length gels and blots with multiple exposures are included in Supplementary Figs. S2–S4): lane 1—Marker, lane 2—the reaction mixture with β2GPI and then TRX (labelled), lane 3—detection of β2GPI using a specific streptavidin HRP enzyme by Western blot, lane 4—unlabelled β2GPI in a streptavidin specific Western blot, lane 5—sample purified by SEC, yielding highly pure labelled protein. (**B**) Peptide intensities obtained by LC–MS/MS analysis from labelled β2GPI. (**C**) Crystal model with the three highest responding peptides highlighted: peptide 10 (sequence 318–326 in DV) in red, peptide 9 (sequence 287–305 in DV) in cyan, peptide 2 (sequence 20–39 in DI) in green. Peptide 287–305 consists of the disulfide partner for peptide TDASDVKPC, suggesting that specific labelling has been achieved.

Reaction mixture was purified and uniform enzymatic reduction and homogenous MPB labelling were confirmed via SDS-PAGE and streptavidin Western blot (Fig. 2A, lanes 2 and 3). Lack of thiols in the untreated protein was further confirmed as no band was identified by streptavidin Western blot (Fig. 2A, lane 4). Purity of reduced species was confirmed by SDS-PAGE (Fig. 2A, lane 5). The biotin quantification assay detected a ratio of 1.0 ± 0.3 biotin molecules per β2GPI molecule, which suggested MPB labelling only on a single thiol.

Specific labelling was confirmed by LC–MS/MS analysis. A total of 10 peptides were identified to contain MPB (Supplementary Table S1) when the protein was digested with trypsin in the native state. All sequence numbers refer to those of the mature sequence as listed in PDB 1c1z, this corresponds to amino acids 20–345 in the immature sequence as stated in Uniprot P02749 and thus, excludes the signaling peptide 1–19. The terminal peptide (peptide 10) containing Cys326 showed the highest response for MPB labelling (Fig. 2B). The peptides with the highest responses were mapped onto a protein model (pdb-ID: 1c1z): peptide 9 (cyan) and peptide 10 (red) contain matching thiols liberated from disulfide bond Cys288/Cys326 (Fig. 2C).

### β2GPI reduction initiates a structural change

**Figure 3:**
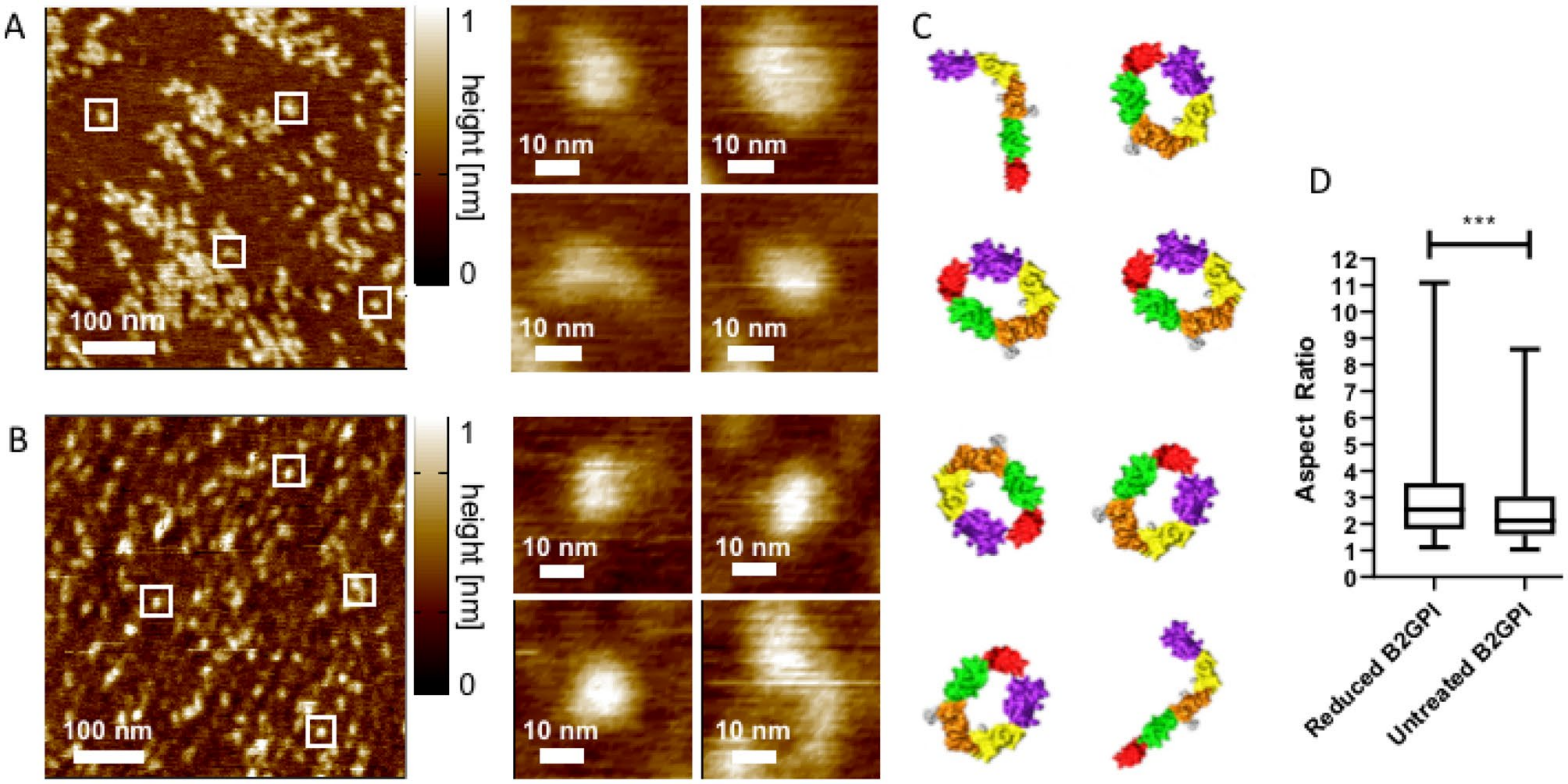
AFM imaging of β2GPI. Untreated (**A**) and reduced (**B**) β2GPI with examples of particle structure highlighted. (**C**) Surface models of β2GPI in the suggested conformation of the structures highlighted in (**A**) and (**B**). (**D**) Distribution of aspect ratios *R* with the minimum and maximum values (whiskers) plotted with the median, this includes three independent replicates of reduced β2GPI and two replicates (with two technical replicates) of untreated β2GPI. In total, 351 and 352 molecules were analysed, respectively. A higher mean aspect ratio for reduced β2GPI (2.97) compared to untreated β2GPI (2.58) and a significantly increased percentage of open particles (35% vs 25%) were found (p = 0.0003). This suggests that reduction allows β2GPI to be more flexible, which is best reflected as a wider upper distribution of structures with more structures falling above an aspect ratio of 5 (10.5% vs 6.3% reduced and untreated, respectively).

Statistical analysis of the conformation of β2GPI was carried out based on AFM imaging as previously published^26^. Images of both reduced and untreated β2GPI (Fig. 3A,B) were analysed for particle shape, which is represented by cartoon images (Fig. 3C).

As defined in the methods section, aspect ratio *R* (particle length/width) above 3 was considered to be open β2GPI, whilst a value lower than 3 was defined as “closed” β2GPI^26^. For both untreated and reduced β2GPI, the majority of molecules were closed, but reduced β2GPI showed a significantly higher proportion of proteins with an aspect ratio > 3 compared to the untreated protein (p = 0.0003).

Although mean aspect ratio was similar between untreated and reduced, the spread of values was higher for reduced β2GPI (Fig. 3D) with increased variance and standard deviation as illustrated in Supplementary Table S2.

We further complemented the AFM measurements with DLS protein size determination analyses which showed a significant shift in hydrodynamic size (Supplementary Fig. S5A). Untreated β2GPI (black) had a hydrodynamic diameter (D_H_) of 13.1 ± 0.7 nm in agreement with previous size determinations^26^. For reduced β2GPI species (red), a significantly smaller D_H_ of 10.1 ± 1.1 nm was found, which was previously associated with a protein conformational change^26,35^.

CD data of untreated (black) and reduced (red) β2GPI showed spectra limited to wavelength above 216 nm due to interfering effects of the biotin function and the HBS buffer (Supplementary Fig. S5B). As can be observed, the CD peaks of reduced β2GPI (red) remain largely within the standard deviations (green and blue) for untreated β2GPI. The slight differences between the two β2GPI species suggested that labelling did not affect the secondary structure and the process has not led to denaturation of β2GPI.

### Modelling studies confirm enhanced flexibility of reduced β2GPI without major change in secondary or tertiary structure

**Figure 4:**
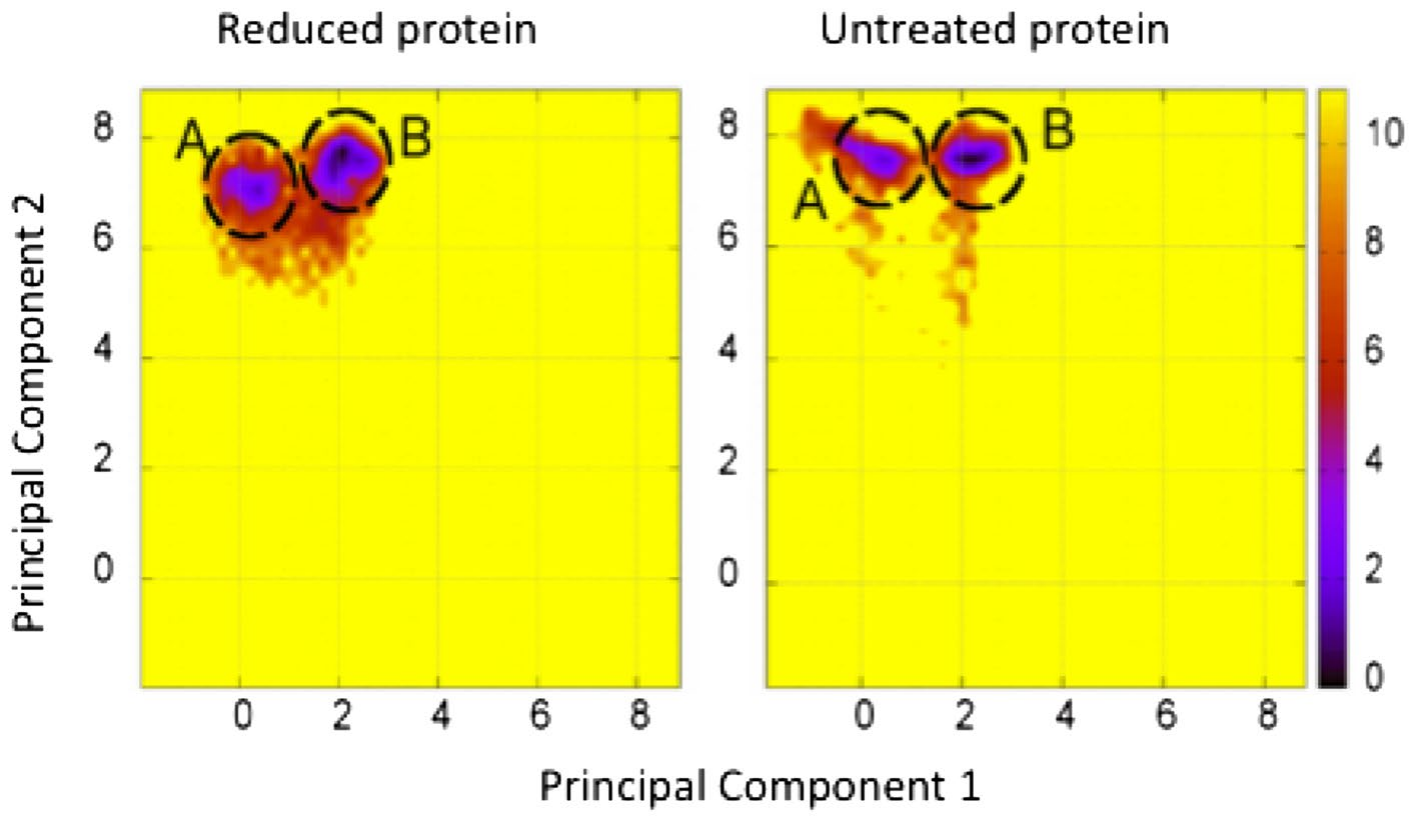
Principal Component Analysis were generated with an in-house code and plotted with GnuPlot 5.4 software (http://www.gnuplot.info) as energy histograms: reduced (left) and untreated (right) β2GPI. The degree of variation is greater in the reduced protein, with larger areas of red seen, whilst the oxidized protein remains fairly stable with smaller areas of red. This suggests greater flexibility in the reduced protein.

A structural model of β2GPI was generated with a reduced disulfide in DV. Later assembly of a closed β2GPI conformation based on affinity analysis was carried out and supplemented with extended carbohydrate chains which stabilized within 70 ns of equilibration. Molecular dynamics simulation (MDS) of reduced *vs* oxidized β2GPI was undertaken for 350 ns and no significant change was seen in tertiary and secondary structure. However, maximal root mean square deviation (RMSD) values between two major groups varied greatly (Fig. 4, spot A and spot B). Reduced β2GPI showed a maximal RMSD of ~ 15 with a range of 4–7, whilst oxidized β2GPI had a much lower range and a maximal value of just 3.6. Nevertheless, A and B are structurally only minorly different with the main deviations being located at the two loop regions at the tip of DV. For both β2GPI forms, structure A is the main population with A:B being 2:1, while the energy barrier between the spots is 1.6 kJ/mol and 1.2 kJ/mol for reduced and oxidized β2GPI, respectively. Both energy barriers are easily overcome by thermal energy making the transition between A and B fast, which suggests an increase in flexibility, with the largest deviations being within DV for reduced β2GPI. This was confirmed by dihedral Principal Component Analysis^36^ which demonstrated increased variation in reduced β2GPI (Fig. 4).

### Reduction of β2GPI increases binding of IgG anti-DI samples from patients with APS

Polyclonal IgG anti-DI antibodies were purified from four triple-positive APS patients, whose characteristics can be seen in Table 1.

**Table 1:** Patient information for the study.

Patient no	Age	Sex	Type of APS	Thrombotic events	Pregnancy morbidity	LA-positive	aCL	Anti-beta2GPI	Anti-DI
1	53	Male	PAPS	1 Arterial	Not applicable	Yes	> 100	86	26
2	57	Female	SLE Associated	Multiple Venous	One termination of pregnancy for medical reasons	Yes	> 100	85	89
3	74	Male	PAPS	Multiple Venous and Arterial	Not applicable	Yes	> 100	> 100	> 100
4	37	Female	SLE Associated	1 Arterial	One first trimester loss, one premature delivery 29/52 due to placental thrombosis	Yes	> 100	> 100	> 100

Demographic and clinical characteristics, as well as antibody levels are given.

*PAPS* primary antiphospholipid syndrome, *SLE* systemic lupus erythematosus, *LA* lupus anticoagulant, *aCL* anti cardiolipin antibodies.

**Figure 5:**
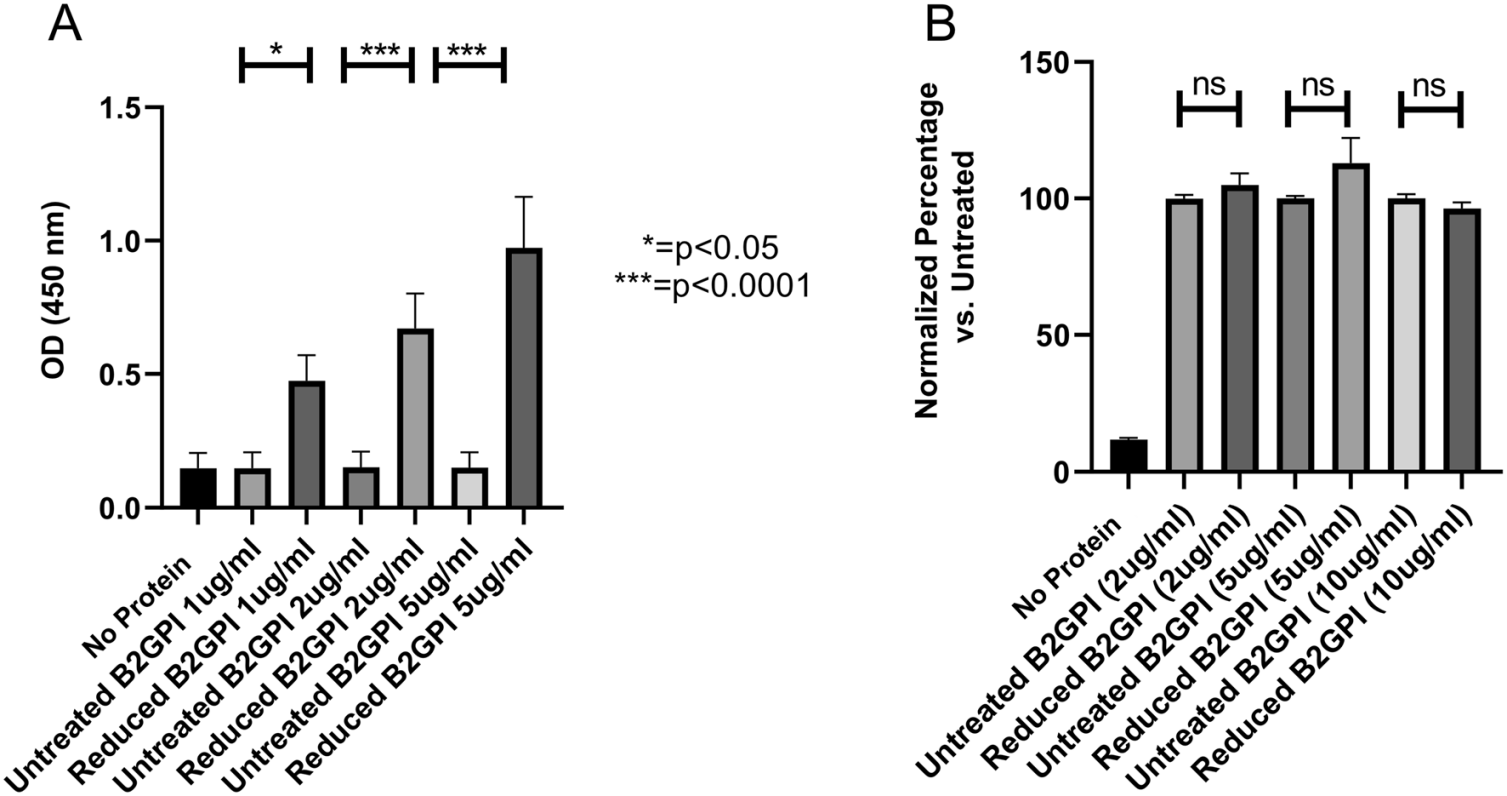
Assessment of antigenicity of reduced β2GPI. Antibodies to DI of β2GPI were purified from serum of APS patients using immunoprecipitation with a proprietary tagged DI molecule on a nickel bead. (**A**) Purified antibodies were coated on a maxisorb plate and incubated with either reduced or untreated β2GPI. Detection occured via a secondary peroxidase-conjugated anti-β2GPI antibody and OD was read at 450 nm. (**B**) Data from the control ELISA with either reduced or untreated β2GPI coated on a maxisorb plate and detected with the same detection system as previously described. As it can be seen, the secondary antibody does not favour detection of the reduced form. The data in Panel B has been normalized, so the effect of untreated β2GPI is considered 100%.

Triple positivity (positive tests 12 weeks apart for anti-Cardiolipin (aCL), anti-Beta-2-Glycoprotein I (aβ2GPI) and Lupus Anticoagulant) is associated with a more severe phenotype for APS patients^36,37^. These polyclonal IgG samples were tested for binding to untreated and reduced β2GPI. Significantly higher binding was seen with reduced protein at all concentrations (Fig. 5A). A dose-dependent response was observed with a maximal concentration of 5 µg/mL reduced β2GPI giving an OD of ~ 1.

A control experiment was carried out to exclude the possibility that the secondary β2GPI-specific antibody showed preferential binding to reduced β2GPI. Figure 5B shows the results of the control ELISA, where the plate was coated directly with both β2GPI species and the β2GPI-specific secondary antibody was added. No difference was seen between binding of secondary antibody to reduced and untreated β2GPI, indicating that the difference in Fig. 5A must be due to differential binding of β2GPI species to the polyclonal patient-derived IgG anti-DI on the plate.

## Discussion

Our results suggests that, although there is a dramatic increase in binding of anti-DI antibodies from APS patients to reduced β2GPI, the structural alteration upon reduction may be more subtle than first hypothesized. This may be explained by an increased flexibility of the molecule, rather than a traditional shift of conformation from closed to open.

Our data challenges several paradigms in the field. It has been assumed by other studies that β2GPI upon purification becomes oxidized and linear. Here, we show that the majority of purified, untreated β2GPI is oxidized but also circular. A second assumption was that antibodies from patients would bind better to oxidized β2GPI due to a structural shift to the open form. However, we found that anti-DI antibodies from patients with APS actually bind more strongly to reduced β2GPI.

Ioannou et al*.* showed that it was possible to label thiols of β2GPI in plasma^15,34^, and proposed that a larger proportion of circulating β2GPI is in the free-thiol form^38^ in healthy controls than in patients with APS^38^. Their suggestion was that increased oxidized β2GPI in these patients exposed DI favouring production of anti-DI antibodies. Raimondo et al*.*^32^ reported increased levels of reduced β2GPI in the serum of anti-DI negative compared to anti-DI positive patients. However, Raimondo and co-workers used an indirect method in which the amount of reduced β2GPI was expressed compared to a pooled healthy control, without correction for increased levels of total β2GPI in APS patients.

Although it had been firmly established in the literature that circulating β2GPI was in the circular conformation^5^, a recent publication by Ruben et al*.*^39^ has presented evidence to the contrary. Importantly, the work by Ruben et al. uses a number of biophysical techniques which have not previously been applied to β2GPI including SAXS and FRET, whilst fomer studies focused on imaging by electron microscopy.

Our work and that of Ruben et al. as being complementary. We used several biophysical techniques in our work, most of which had also not been utilized frequently before to study β2GPI. Our findings differ from those of Ruben and colleagues and there are a number of possible reasons for this, apart from the use of different biophysical techniques. The source of β2GPI for the work of Ruben et al*.* was purified expressed β2GPI from cellular systems rather than from healthy or APS patient serum as shown in the previous studies^5,29,40^.

We used commercial β2GPI, which is obtained from healthy human serum and therefore, a direct comparison of our results with Ruben’s results is complicated.

Interestingly, a recent paper by Szabo et al.^41^ also carried out imaging and binding studies of β2GPI purified by perchloric acid precipitation method which behaved similarly to β2GPI closed by pH gradient (confirmed by electron microscopy), whilst “opened” β2GPI (confirmed by electron microscopy) had significantly different effects in clotting assays. This contrasts with the work of Ruben et al*.*^39^ who show control β2GPI (unmodified) behaves similarly to the proven open expressed β2GPI.

Similarly, Szabo et al.^41^ show that the open or closed β2GPI have comparable binding affinity to purified anti-β2GPI antibodies, suggesting again that antibody binding might be structurally independent. This highlights the somewhat contrasting results within the field obtained by authors using diverse techniques.

Ioannou et al. used a different method to demonstrate free thiols in serum β2GPI from healthy subjects (not patients with APS) including binding to a streptavidin plate and using a murine anti-β2GPI^29^. Our work represents the first study of specifically reduced β2GPI and its binding to purified pathogenic anti-DI antibodies derived from patients with APS.

The effect of MPB cannot be discounted as it may give a steric effect on the opening of the molecule and it may also influence stability or purification. Biotin is known to interfere in circular dichroism spectroscopy below a wavelength of 215 nm; as such we excluded this region from our spectra and focused on the 216–270 nm region. We also limited these effects by titrating the MPB concentration used and confirmed limited non-specific conjugation through LC–MS/MS analysis. Similarly, the presence of reduction in DI, which was previously published^33,40,42^, raises the possibility that the increased autoantibody binding seen is due to reduction in DI rather than DV. Although this is difficult to refute totally, the biotin quantification assay showed only one thiol per molecule and LC–MS/MS showed that the majority of molecules were labelled in the C-terminal peptide (Cys326). Therefore, the major change in antibody-binding we observed is most unlikely to be due to reduction in DI.

As discussed, the combination of LC–MS/MS and biotin quantification suggests that our system specifically labels the terminal disulfide Cys288/Cys326 of β2GPI. Labelling of the single thiol rather than both the thiols of the terminal disulfide may be due to the lack of solvent accessibility for Cys288, the partner of Cys326 (the C-terminal thiol). It should be highlighted, however, that the LC–MS/MS and biotin quantification cannot discount heterogeneity of the sample and thus, the inclusion of multiple labelled species and unlabelled species in minor amounts.

Molecular dynamics simulations showed no significant conformational shift upon reduction, which is consistent with our other data. Interestingly, however, a hydrogen bond near the terminal disulfide stabilized the circular form of β2GPI. This is partially confirmed by reduced β2GPI having an increased flexibility of the loop regions close to the bond that allows for the disruption of this hydrogen bond. Nevertheless, further experimental work for validation is required.

Interestingly, our data suggest that, instead of a simple dichotomy between open and closed structures, the structural spectrum of β2GPI may be more subtle with ranges of flexibility.

Although DLS shows significant structural shifts from the non-modified protein, size measurements might be influenced by addition of MPB ligand which alters the electrophoretic properties of the protein. In addition, particle sizing methods can describe accurately spherical particles, but are limited regarding the size determination of non-spherical particles. A change in the lenght of non-spherical particles will impact the size, while a change in the diameter will be hard to detect (the diffusion will be barely influenced). DLS results suggest a more elongated character in reduced β2GPI, as the hydrodynamic radius is smaller. This is consistent with the higher number of molecules with aspect ratio > 3 in the AFM results.

CD spectroscopy results may confirm our theory for increased flexibility. However, due to HBS buffer interferences and strong interference of the MPB ligand (at least at wavelength below 215 nm) in the CD spectra, it is difficult to assess whether the differences in the CD spectra of the untreated and reduced β2GPI are significant or not.

The striking change in activity towards APS autoantibodies between the reduced and untreated protein species could also be explained by increased flexibility in DV post-reduction, allowing the R39–R43 epitope in DI to become accessible in the fluid phase despite the molecule presenting an overall circular conformation.

Our study attempts at thoroughly associating the structure of reduced β2GPI with its function. The results show a clear change in binding to anti-DI from patients with APS after reduction, potentially explained by increased flexibility in DV. This has significant implications for how these pathogenic antibodies may respond in sites of oxidative stress where β2GPI is structurally flexible and thus more readily able to bind anti-DI antibodies. Through the use of structural techniques we refute several paradigms within the field including the idea that oxidized β2GPI must be linear. This refutation may have wide ranging implications for both our understanding of the molecule and, given the alteration in antibody binding, the pathogenesis of APS itself. Further studies are required to elucidate the relationship between function and structure in reduced β2GPI.

## Methods

### Ethics

This study was carried out in accordance with recommendations of London Hampstead Research Ethics Committee Ref No 12/LO/0373 with written informed consent from all subjects in conformity with the Declaration of Helsinki. The experimental protocol was approved by the London Hampstead Research Ethics Committee Ref No 12/LO/0373.

### Reagents

Unless otherwise stated, all chemicals were purchased from Sigma. Human plasma β2GPI was bought from Enzyme Research Laboratories. Protein concentration was determined using a bicinchoninic acid assay.

### Ellman’s reagent assay

To determine free thiol concentration, 35 µM untreated β2GPI and samples for a cysteine hydrochloride monohydrate standard curve (0–1.5 mM) were prepared in reaction buffer (100 mM sodium phosphate, 1 mM EDTA, pH 8.0). 4 mg/mL Ellman’s reagent (Thermo Fisher, Darmstadt, Germany) was also dissolved in reaction buffer. A mixture consisting of 200 µL reaction buffer, 4 µL Ellman’s reagent and 20 µL sample or cysteine standard was added in a 96 well plate (Sarstedt, Nümbrecht, Germany) and incubated for 15 min at room temperature (RT) followed by absorption measurement at 412 nm.

### SDS-PAGE and western blot analysis

For SDS-PAGE, samples were treated under non-reducing conditions and loaded to 4–12% gradient bis–Tris SDS gels (Thermo Fisher). Gels were stained with SimplyBlue SafeStain (Thermo Fisher) or used for semidry Western blot transfer (30 min, 15 V) to a nitrocellulose membrane (Amersham Protran 0.2 µm NC). Membrane was blocked with 5% milk powder in TBS (50 mM Tris, 0.15 M NaCl, pH 7.4) containing 0.05% Tween20 (TBST) for 1 h at RT and washed three times before incubation for 30 min at RT with peroxidase-conjugated streptavidin (Jackson ImmunoResearch) in 5% milk powder/TBST. After three additional washing steps, the membrane was stained with peroxidase substrate (West Pico Chemiluminescent Substrate, Thermo Fisher).

### β2GPI reduction

All reaction steps were performed under argon atmosphere at 25 °C. 50 µM recombinant human thioredoxin-1 (TRX-1) was reduced by 50 µM TCEP (Tris(2-carboxyethyl)phosphine hydrochloride) in a total volume of 100 µL Hepes-buffered saline (HBS; 20 mM Hepes, 140 mM NaCl, pH 7.4) for 1 h with mild shaking. 900 µL HBS buffer and 0.2 µM β2GPI were added and incubated for 1 h at with mild shaking. 15 µM MPB (N^a^-(3-maleimidylpropionyl)biocytin; Thermo Fisher) were added for 15 min with mild shaking to label free thiols. Finally, 200 µM reduced glutathione (GSH; Thermo Fisher) was used to quench unbound MPB (15 min). For purification, the reaction mixture was first volume-reduced by a 10 kDa cut-off centrifugal filter unit (Merck) at 2500 × *g*, followed by size exclusion chromatography (SEC) with a Superdex 75 Increase column (GE Healthcare) with a flow rate of 0.25 mL/min in HBS buffer. β2GPI main peak SEC fractions were pooled and concentrated using centrifugal filters with a cut-off of 10 kDa (Merck) at 4000×*g*.

### LC–MS/MS sample preparation and analysis

For analysis of peptides of MPB labelled β2GPI, 4 µg of total protein from each sample were mixed with 20 mM ammonium bicarbonate, trypsin (Promega) was added at a ratio of 1:25 and samples were incubated overnight at 37 °C. Tryptic digestion was stopped through addition of acetic acid to a final concentration of 1% followed by desalting (ZipTip-µC18 tips, Merck-Millipore). Extracts were concentrated by evaporation under vacuum and subsequently resolved in 0.1% acetic acid, 2% acetonitrile (ACN). LC–MS/MS analysis was performed as described previously^43^ with minor modifications. Chromatographic separation of tryptic peptides was achieved on a reverse phase nano-Acquity UPLC column (1.7 μm, 100 μm i.d. × 100 mm, Waters GmbH) using a 50 min non-linear gradient from 2 to 60% ACN in 0.1% acetic acid (flow rate: 400 nL/min). The nano-LC column was interfaced using electro-spray-ionization (ESI) to a LTQ-Orbitrap Velos mass spectrometer (Thermo Scientific). Precursor ions of a mass over charge range (m/z) 325–1525 (r = 30.000) were subjected to data dependent MS/MS fragmentation of Top-20 peaks in the ion trap at a collision induced energy (CID) of 35%. Repetitive MS/MS acquisition was avoided by setting dynamic exclusion of 60 s for already selected precursors.

Data analysis was carried out using Proteome Discoverer 2.2 software (Thermo Scientific). Peptides were identified by search against the Uniprot human database (rel. 2017_01) using the following settings: trypsin as cleavage enzyme, 2 missed cleavage, oxidation on methionine, and biotin:Invitrogen-M1602 on cysteine residuals were selected as dynamic modifications. Only peptides identified with a false discovery rate (FDR) < 1% were used for further analysis. These peptides also demonstrated XCorr values > 2.0.

### Quantification of MPB labelling

To quantify the amount of MPB labelling per β2GPI molecule, a biotin quantification assay (colorimetric biotin assay kit, Sigma) was applied. 180 μL of a 4′-hydroxyazobenzene-2-carboxylic acid (HABA)/avidin assay mixture and 20 μL of 9–14 μM reduced β2GPI sample, 20 μL HBS buffer (negative control), or 20 μL of 100 μM biotin in ddH2O (positive control) were added to microtiter plate wells (Sarstedt). The reaction mixture was incubated for 5 min at RT with mild shaking (150 rpm) in the dark and the absorbance was measured at 500 nm. The biotin concentration was calculated according to the manufacturer’s specifications using the extinctions coefficient of the HABA/avidin complex.

### Atomic force microscopy (AFM)

AFM imaging of untreated as well as reduced β2GPI was executed on a Veeco NanoScope V with a JV-scanner (Bruker, Billerica, MA, USA). Images were recorded in AC/tapping mode in air using a PointProbe Plus AFM cantilever with a tip radius of curvature of < 10 nm and a spring constant of 42 N/m (Nanosensors). A resonance frequency of approximately 330 kHz, as well as a sampling rate of 512 × 512 pixels with a scan rate of 1.0–1.5 Hz, were applied. For AFM sample preparation, (untreated and reduced) β2GPI solutions were diluted to a concentration of 2 μg/mL in HBS buffer. 60 μL of each sample was dropped on a freshly cleaved mica sheet (Electron Microscopy Sciences) and incubated for 5 min to allow protein adsorption. The excess solution was carefully removed from the edge of the mica sheet with a lint-free paper and the unbound protein was taken away from the surface by soaking the mica sheet in deionized water for 30 s. Further, the water was removed with a lint-free paper and mica sheets were dried in a laminar flow box for 30 min.

### AFM image analysis

Analysis of β2GPI conformation from AFM imaging was done as we previously described^26^. Briefly, the aspect ratio *R* (length/width) of single, flatly adsorbed β2GPI particles was calculated from AFM images. β2GPI in closed conformation were identified by an aspect ratio between *R* = 1 and *R* = 3. In contrast, β2GPI in open conformation led to a typical aspect ratio between *R* = 3 and *R* = 10. Hence, a threshold value of *R* = 3 was chosen to distinguish between β2GPI in open and closed conformation in automated script analysis. *R* value data for each analyzed β2GPI particle were represented as box plots. The quantiles of the box refer to 25 and 75%, whereas the whisker includes minimum and maximum of the population. The percentage values of closed and open protein conformation were determined from the total number of analyzed particles.

### Circular dichroism (CD) spectroscopy

CD measurements in the far-UV (215–270 nm) were carried out with a Chirascan CD spectrometer (Applied Photophysics) at 25 °C using a protein concentration of 1 μM in HBS buffer in 5 mm path length cuvettes. CD spectra acquired with a bandwidth of 1.0 nm, a scanning speed of 15 nm/min and five repetitions were blank corrected and the wavelength dependent mean residue delta epsilon (MRDE) was calculated to normalize data for concentration, number of amino acids and path length of the cuvette.

### Dynamic light scattering (DLS)

DLS measurements were carried out in HBS buffer using a Zetasizer Nano-ZS (Malvern Instruments). 120 μL of sample (6 μM β2GPI) previously vacuum-degassed for 20 min at 25 °C were transferred to 10 mm path length cuvettes, equilibrated to 25 °C for 5 min and measured 10 times (20 runs with run duration of 10 s each) at a detector angle of 173°. Hydrodynamic diameter (D_H_) data, using refractive index of 1.45 and absorption of 0.001 with standard solvent parameters as referred to water, was analyzed with Zetasizer 7.11 software was displayed as intensity *vs.* diameter size plot normalized to 100% intensity.

### Purification of anti-DI IgG

Antibodies were purified as previously described^20^. Briefly, 50 µL of 1 mg/mL Domain I (DI) was immobilized on nickel beads (100 µL) for 30 min at RT on a rotating plate. Patient serum (300 µL) was added (diluted 1:1 in 100 mM sodium phosphate buffer saline (PBS), pH 7.4) and left to bind for 1 h rotating. Beads were washed with 100 mM sodium phosphate buffer (3 × 1 mL) and bound protein was eluted using 0.1 M glycine (pH 2.3). The eluate was dialysed against 100 mM sodium phosphate, 150 mM NaCl, pH 7.2 and purified across a protein G column (GE Healthcare) before elution in 0.1 M Glycine (pH 2.3) and dialysis to PBS for quantification. All patients were triple positive (a severe phenotype with exacerbated disease).

### ELISA

ELISA procedure was adapted according to Agar et al*.*^5^. A microtiter plate (MaxiSorp Nunc-Immuno, Thermo Fisher) was coated with 100 µL/well of either 5 µg/mL anti-DI antibody or 50 mM carbonate buffer, pH 9.6 at 4 °C overnight. The plate was washed with TBS containing 0.1% Tween20 and blocked with 200 µL/well 3% bovine serum albumin (BSA) in TBS for 2 h at 37 °C. After washing the plate three times, 0, 1, 2, or 5 µg/mL β2GPI species were incubated in 0.3% BSA/TBS (100 µL/well) for 1 h at RT. The plate was washed three times and 100 µL per well of peroxidase-conjugated anti-β2GPI antibody at 1 µg/mL (Affinity Biologicals) diluted in 0.3% BSA/TBS was added for 1 h at RT. After three final washing steps, 100 µL/well TMB (BD Biosciences) was added, reaction was stopped by 100 µL 1 N H_2_SO_4_ after 15 min and absorption measured at 450 nm. Statistical significance was derived by T-Tests and ANOVA using Graphpad Prism 8.

### Control ELISA

Microtiter plates were coated with 50 μL/well of 2, 5, or 10 μg/mL β2GPI (either untreated or reduced) in TBS for 1 h at 37 °C. TBS alone was coated as a negative control. Plates were washed three times and blocked with 200 μL/well 3% BSA/TBS for 2 h at 37 °C. The plate was washed again three times, before addition of 50 μL/well 1 μg/mL peroxidase-conjugated anti-β2GPI antibody in 0.3% BSA/TBS for 1 h at RT. After three final washing steps, substrate was applied and absorption was measured at 450 nm.

### Modelling

To supplement the results of the structural experiments we carried out computer simulations of the effect of reduction at Cys288/Cys326 on the structure of β2GPI. Simulations were performed with GROMACS 5.1 software package^44^ using the AMBER99SB-ildn force field^45^. By increasing the hydrogen mass to 4 m.u. and reducing the corresponding heavy atom mass by the same amount (hydrogen mass repartitioning)^46^, the verlet integrator^47^ was applied only every 7 femtoseconds. The explicit water was represented by the TIP3P model^48^ and carbohydrates with the Glycam force field^49^. Periodic boundary conditions were used together with the removal of the overall center of mass motion. Intermolecular Coulomb and van der Waals interactions were considered using cutoffs of 1.0 nm with switching functions at 0.9 nm. Long-range electrostatic interactions were calculated with the PME method^50^ using a grid spacing of 1.2 nm. All bonds were constraint to the optimal distance with the LINCS algorithm^51^. Temperature was controlled separately for protein and non-protein to 300 K every 100 femtoseconds using a modified v-rescale thermostat^52^. A Parrinello–Rahman barostat^53^ adjusted the pressure to 1 bar every 12 picoseconds. All simulations were visualized with VMD 1.9.211^54^.

## Supplementary Information

Supplementary Information.
