# Supplementary material for: Specific domain V reduction of beta-2-glycoprotein I induces protein flexibility and alters pathogenic antibody binding
Source: Sci Rep. 2021 Feb 25;11:4542. doi: 10.1038/s41598-021-84021-2 (PMC7907366; doi:10.1038/s41598-021-84021-2)
Supplement: Supplementary file 1 — Supplementary Information. [file 41598_2021_84021_MOESM1_ESM.pdf]

Supplementary Information

Specific domain V reduction of beta-2-glycoprotein I induces protein flexibility and alters pathogenic antibody binding

Ina Buchholz[1,2 a], Thomas McDonnell[3 a], Peter Nestler[4], Sudarat Tharad[5], Martin Kulke[1], Anna Radziszewska[6], Vera M. Ripoll[7], Frank Schmidt[8,9], Elke Hammer[8,10], Jose L. Toca-Herrera[5], Anisur Rahman[7 *] and Mihaela Delcea[1,2,10 *]

[1]*Institute of Biochemistry, University of Greifswald, Germany*
[2]*ZIK HIKE, Fleischmannstr. 42, 17489 Greifswald, Germany*
[3]*Division of Biochemical Engineering, Bernard Katz Institute, University College London, UK*
[4]*Institute of Physics, University of Greifswald, Germany*
[5]*Institute for Biophysics, University of Natural Resources and Life Sciences Vienna, Austria*
[6]*Centre for Adolescent Rheumatology, Division of Medicine, University College London, UK*
[7]*Centre for Rheumatology, Division of Medicine, University College London, UK*
[8]*Interfaculty Institute for Genetics and Functional Genomics, University of Greifswald, Germany*
[9]*Proteomics Core, Weill Cornell Medicine-Qatar, Doha, Qatar*
[10]*DZHK (German Centre for Cardiovascular Research), partner site Greifswald, Greifswald, Germany*

[a] *These authors contributed equally to this work.*

*Corresponding authors: anisur.rahman@ucl.ac.uk and delceam@uni-greifswald.de

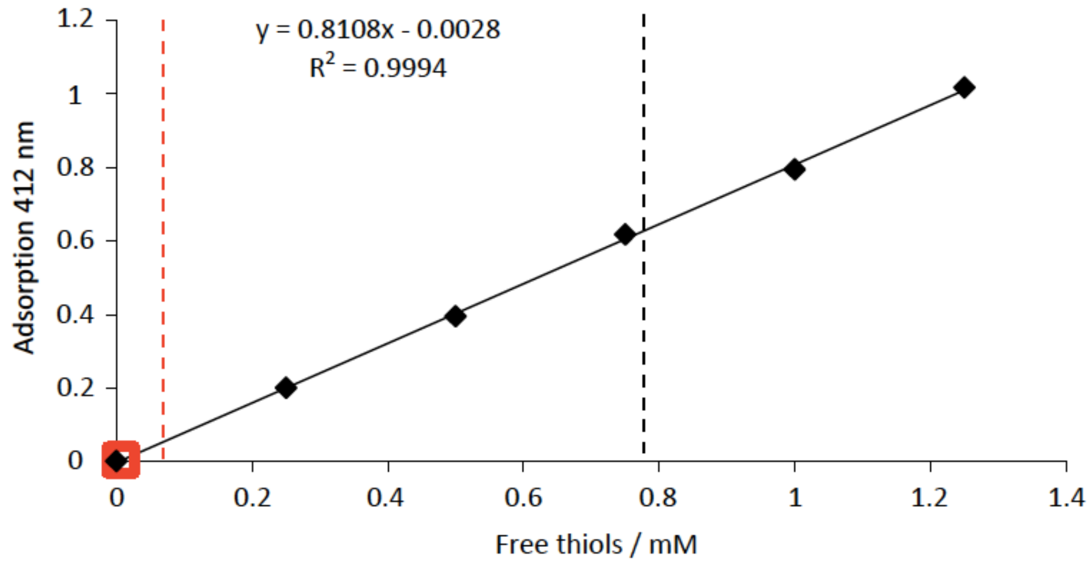

Supplementary Figure S1. Ellman's reagent assay for the determination of free thiols in untreated β2GPI. The dashed red line indicates the expected concentration of free thiols if just one disulfide was reduced, whilst the black dashed line represents all 22 potential free thiols in β2GPI. The red square highlights the values gained for untreated β2GPI suggesting that no free thiols are present in untreated β2GPI.

Supplementary Table S1. Detection of MPB labelled peptides of β2GPI. XCorr values are shown to highlight the high confidence of peptide identification with values > 2.0; aa = amino acids.

Peptide #	aa	Peptide Sequence	Theoretical Mass [Da]	Peptide intensity ($x10^3$)	XCorr
1	*3 - 19*	T**C**PKPDDLPFSTVVPLK	2380.19	119	4.1
2	*20 - 39*	TFYEPGEEITYS**C**KPGYVSR	2849.28	500	2.4
3	*44 - 59*	(K)FI**C**PLTGLWPINTLK	2367.26	256	3.9
4	*64 - 77*	V**C**PFAGILENGAVR	1968.97	292	4.1
5	*78 - 104*	YTTFEYPNTISFS**C**NTGFYLNGADSAK	3534.55	155	3.4
6	*105 - 110*	**C**TEEGK	1189.49	94	2.1
7	*186 - 208*	**C**PFPSRPDNGFVNYPAKPTLYYK	3197.52	99	4.1
8	*211- 231*	ATFG**C**HDGYSLDGPEEIE**C**TK	3318.37	85	4.4
9	*287 - 305*	(K)**C**SYTEDAQ**C**IDGTIEVPK	3146.38	634	3.5
10	*318 - 326*	TDASDVKP**C**	1458.62	4935	3.6

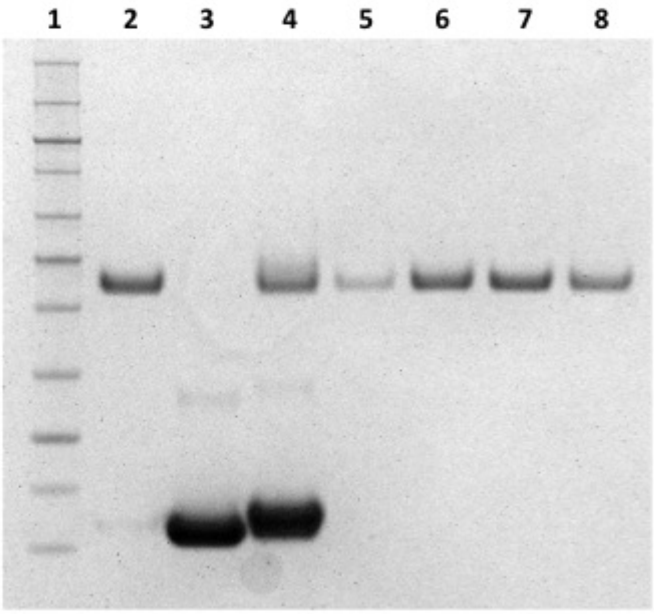

Supplementary Figure S2. Non-reductive SDS-PAGE gel: lane 1- Marker, lane 2- untreated β2GPI, lane 3- TRX-1 with TCEP (control), lane 4- reaction mixture after reduction with β2GPI and TRX (labelled), lane 5- reduced β2GPI after purification by SEC (1:1 dilution), lane 6- β2GPI with MPB/GSH (control), lane 7- β2GPI with MPB (control), lane 8 - reduced β2GPI after purification by SEC (no dilution).

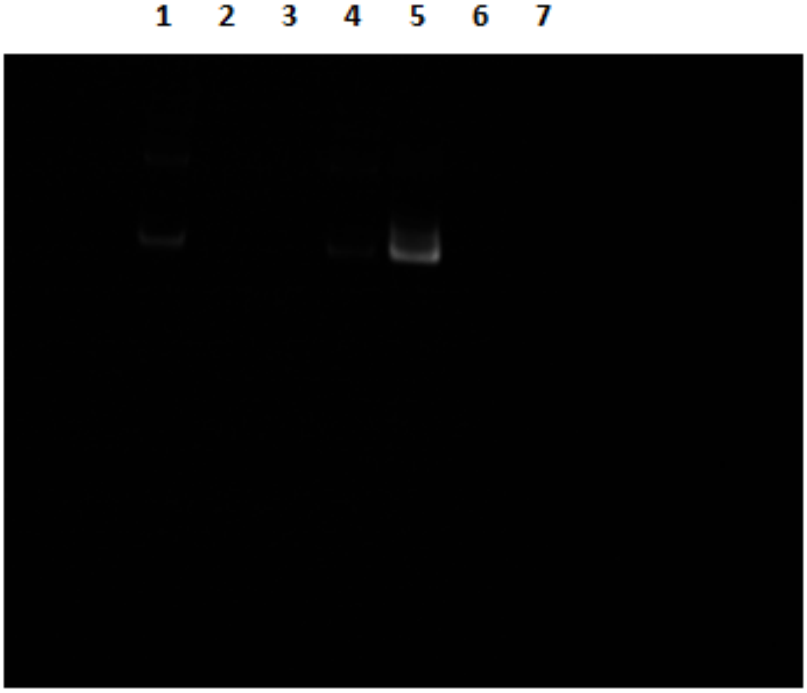

Supplementary Figure S3. Western Blot with moderate exposure time corresponding to the non-reductive SDS-PAGE gel shown in Figure S2. Lane 1- reduced β2GPI after purification by SEC (no dilution), lane 2- β2GPI with MPB (control), lane 3- β2GPI with MPB/GSH (control), lane 4- reduced β2GPI after purification by SEC (1:1 dilution), lane 5- reaction mixture after reduction with β2GPI and TRX (labelled), lane 6- TRX-1 with TCEP (control) and lane 7- untreated β2GPI.

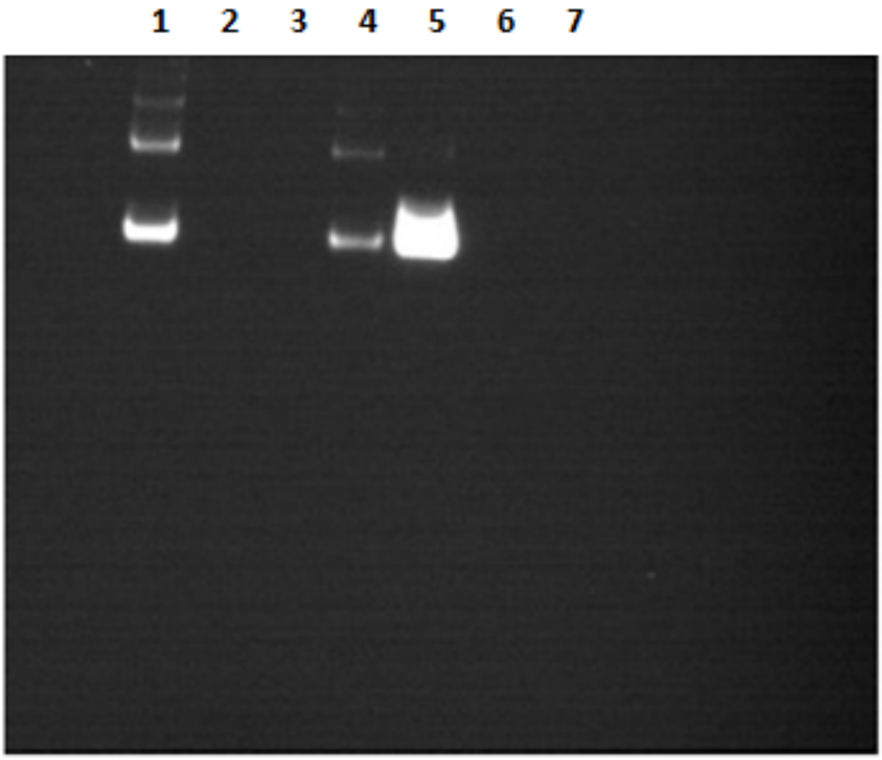

Supplementary Figure S4. Western Blot with most extended exposure time corresponding to the non-reductive SDS-PAGE gel shown in Figure S2. Lane 1- reduced β2GPI after purification by SEC (no dilution), lane 2- β2GPI with MPB (control), lane 3- β2GPI with MPB/GSH (control), lane 4- reduced β2GPI after purification by SEC (1:1 dilution), lane 5- reaction mixture after reduction with β2GPI and TRX (labelled), lane 6- TRX-1 with TCEP (control) and lane 7- untreated β2GPI.

Supplementary Table S2. Statistical shape analysis of the AFM data. The percentage of proteins with an aspect ratio $R > 3$, i.e. in open conformation is significantly higher in reduced protein (35% vs 25%, non-parametric T Test, $p = 0.0003$). The two species also have significantly different distributions (K-S test, $p = 0.0014$) as shown by the difference in variance (2.92 vs 1.94) suggesting that there is a more subtle change in structure based around flexibility upon reduction of β2GPI in addition to an increase in the proportion in the open conformation.

**Variable**	**TRX-1 reduced β2GPI**	**Untreated β2GPI**
Total molecules analysed	351	352
R above 3 (%)	35.33	25.00
Mean	2.97	2.58
Median	2.55	2.13
Max	11.10	8.59
Min	1.11	1.04
SD	1.71	1.39
Variance	2.92	1.94

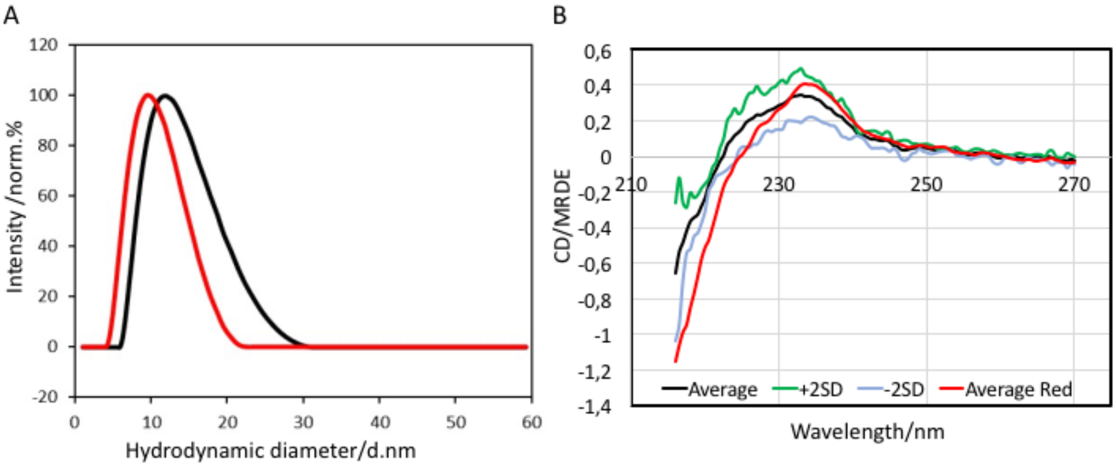

Supplementary Figure S5. (A) Size distribution of untreated (black) *versus* reduced (red) β2GPI measured by DLS showed a decrease in hydrodynamic diameter upon β2GPI reduction. (B) CD spectra of untreated (black, average of n = 3) and reduced (red, average of n = 4) β2GPI. The standard deviation (SD) at each wavelength was calculated for untreated protein and curves have then been plotted in both directions: mean plus 2xSD (green) and mean minus 2xSD (blue).
